# Long COVID: Lung Pathophysiology and its Relationship with Cognitive Dysfunction

**DOI:** 10.21203/rs.3.rs-7464124/v1

**Published:** 2025-10-08

**Authors:** Keegan R Staab, Marrissa J McIntosh, Abhilash S Kizhakke Puliyakote, Andrew D Hahn, Natally Alarab, Jonathan L Percy, Tara Lanning, Johanna Theeler, Carinda Linkenmeyer, Conner J Wharff, Eric Bruening, Jessica C. Sieren, Eric A. Hoffman, Alejandro P. Comellas, Karin F. Hoth, Sean B. Fain

**Affiliations:** University of Iowa; University of Iowa; University of Iowa; University of Iowa; University of Iowa; University of Wisconsin – Madison; University of Iowa; University of Iowa; University of Iowa; University of Iowa; University of Iowa; University of Iowa; University of Iowa; University of Iowa; University of Iowa; University of Iowa

## Abstract

Post-acute sequelae of COVID-19 (Long COVID) includes physical and cognitive symptoms that can last long after acute infection. Links between lung pathophysiology and cognitive dysfunction in Long COVID remain largely unexplored. Long COVID participants were recruited from a post-COVID-19 clinic. Participants completed Patient-Reported Outcomes Measurement Information System (PROMIS) symptom questionnaires for Sleep Disturbance, Anxiety, Depression, and Cognitive Function, the National Institute of Health Toolbox Cognition Battery (NIHTB-CB), pulmonary function tests (spirometry, diffusion capacity of the lung), structural and functional brain magnetic resonance imaging (MRI), and 129 Xe MRI for ventilation and regional pulmonary gas exchange evaluation, at the same study visit. Bivariate relationships between lung and cognitive function in Long COVID were assessed using Spearman partial correlations, adjusted for age. Twelve participants (age=54±11 yrs.; 10 females) that were 32±5 months from infection were evaluated. PROMIS symptom scores indicated reduced perceived cognitive function in everyday life along with increased fatigue, anxiety, depressive symptoms, and sleep disturbance. However, objective cognitive function performance on NIHTB-CB were broadly within normal limits. Lower 129 Xe MRI gas exchange was correlated with more severe symptoms of sleep disturbance, reduced executive functioning performance, and elevated cerebral perfusion via brain MRI. These results are suggestive of a link between lung pathophysiology and cognitive dysfunction in this Long COVID population with enduring respiratory and cognitive symptoms more than two years after infection.

## INTRODUCTION

A substantial portion of individuals recovering from acute COVID-19 continue to experience persistent COVID-related symptoms well beyond the resolution of the initial infection. These symptoms are highly heterogeneous and affect multiple organ systems^1,2^. The most common symptoms include dyspnea, fatigue, exercise intolerance and brain fog^1,3^. The persistence of symptoms for more than three months is broadly called Long COVID and, as recent evidence suggests, can last for years^1^. In the United States, Long COVID is estimated to affect approximately 14% of those initially infected with SARS-CoV-2^4^, though some estimates suggest the prevalence to be as high as 60%, impacting millions of people^5^.

Dyspnea is one of the most prevalent symptoms of Long COVID, but unfortunately, traditional pulmonary function testing does not detect significant pulmonary dysfunction in most of these patients complicating both diagnosis and management. Hyperpolarized xenon-129 (^129^Xe) magnetic resonance imaging (MRI) is a technique which allows for the sensitive, regional measurement of lung function, including gas exchange. This is possible because ^129^Xe can freely diffuse into the lung parenchyma and blood plasma, and red blood cells (RBC) with distinct chemical shift frequencies on MRI. ^129^Xe MRI has been used in several Long COVID studies to characterize ventilation and gas exchange^6–13^, revealing that ^129^Xe has sufficient sensitivity to detect lung function abnormalities associated with Long COVID. Longitudinal studies further indicate that while some improvement in ^129^Xe MRI measures occurs over time, abnormalities may persist for up to a year post infection^8,13^.

In addition to respiratory symptoms, approximately 40% of Long COVID patients report brain fog such as memory complaints and trouble concentrating^14^. Brain MRI is used as a tool to study these cognitive and central nervous system manifestations. Results have varied between studies, however a review article of over 1200 Long COVID participants from 25 studies that used brain MRI highlighted gray matter (GM) atrophy, changes in diffusion weighted imaging, and a reduction in perfusion to be the most consistent findings^15^.

Despite the clear respiratory involvement in acute COVID-19 and the persistence of both respiratory and cognitive symptoms in Long COVID, the relationship between pulmonary and neurological abnormalities remains poorly understood. To date, no prospective studies have systematically investigated the links between lung and brain dysfunction in this population. We have three primary hypotheses: 1) This Long COVID sample will have lower measures of gas exchange compared to the control population, 2) There is a link between pulmonary and cognitive function such that metrics of ^129^Xe pulmonary gas exchange will correlate with objective cognitive function in people with Long COVID, and 3) ^129^Xe MRI measures of lung function will correlate with brain MRI measures of volume, perfusion, and white matter (WM) integrity.

## RESULTS

### Study Sample

Twelve participants (10 females; age=54±11 years, range=30–64 years) and ten healthy controls (8 females; age=47±12, range=28–65) were evaluated. Long COVID participants were 32±5 months (range=24–43 months) from acute COVID-19 infection. One participant was omitted from National Institute of Health Toolbox V3 Cognition Battery (NIHTB-CB) analysis due to non-credible performance validity identified using the EVI criteria described in the methods. Due to time constraints during study visits, not all neuroimaging scans were acquired for every participant. Diffusion tensor imaging (DTI) was obtained for 9 out of 12 participants and arterial spin labeling (ASL) was acquired for 10 out of 12 participants. However, structural brain scans were obtained for all participants.

### Demographics, Medical History and Lung Measures

Cohort demographics and pulmonary function are shown in Table 1. Long COVID participants and healthy controls were well-matched for age (p=0.17), sex (p=0.85), and BMI (p=0.36). A summary of pre-existing comorbidities and Long COVID symptoms are reported in Table 2. Of the participants with a complete medical record, reported pre-existing comorbidities include hypertension (3/11), asthma (3/11), fibromyalgia (2/11), depression (2/11), neuropathy (2/11), diabetes mellitus (1/11), and sleep apnea (1/11). Each participant had a minimum of two symptoms that can be attributed to Long COVID with the most reported symptom being fatigue (8/11), followed by dyspnea (6/11), and headache (5/11). All participants had normal PFTs except one Long COVID participant who had a normal FVC (>80 percent predicted) but abnormal FEV1, FEV1/FVC, FEF25–75, and DLCO (all ≤70 percent predicted). There were no significant differences between Long COVID participants and healthy controls on PFT measures or ^129^Xe pulmonary MRI (all p>0.35).

### Symptom Questionnaires and Objective Cognitive Functioning

**Figure 1:**
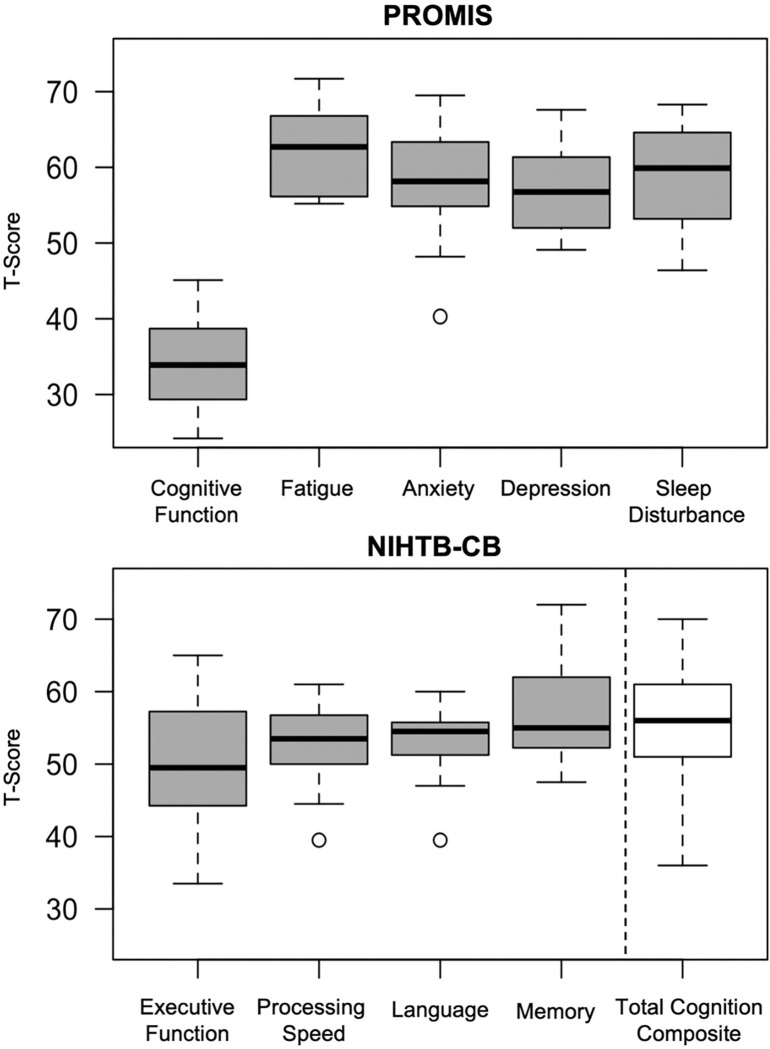
Box and whisker plots for objective and subjective cognitive assessments Top: PROMIS t-scores indicating decreased cognitive function (33.9[29.6–38.7]) as well as elevated fatigue (62.7[56.6–66.8]), anxiety (58.2[55.2–63.3]), depression (56.8[52.0–60.9]), and sleep disturbance (59.9[53.4–64.1]). Bottom: NIHTB-CB age and education adjusted t-scores indicating slightly decreased executive function (49.8[44.3–57.3]), and slightly increased processing speed (52.5[50.0–56.8]), language (52.8[51.3–55.8]), memory (57.0[52.3–62.0]), and total cognition composite scores (55.5[51.0–61.0]). All values reported as (median [1^st^ quartile-3^rd^ quartile]). Abbreviations: PROMIS=Patient Report Outcomes Measurement Information System; NIHTB-CB=National Institute of Health Toolbox Cognition Battery

Box and whisker plots of t-scores for Patient-Reported Outcomes Measurement Information System (PROMIS) symptom questionnaires and NIHTB-CB are shown in Figure 1. Mean PROMIS scores (**Table S1**) for participants with Long COVID indicated significantly reduced perceived cognitive function in everyday life (μ_t-score_=33.9, p<0.001), and elevated symptoms of fatigue (μ_t-score_=62.7, p<0.001), anxiety (μ_t-score_=58.2, p=0.005), depression (μ_t-score_=56.8, p=0.001), and sleep disturbance (μ_t-score_=59.9, p<0.001), but average dyspnea severity (μ_t-score_=47.2, p=0.33), and pain interference (μ_t-score_=53.9, p=0.28) relative to normative expectations for healthy individuals. In contrast to the subjective report of significant cognitive difficulties in everyday life, the participants’ performance on objective NIHTB-CB tasks showed that ten of eleven participants had total cognitive composite scores that were within normal limits (i.e., within one standard deviation of the mean, or better). The single participant with total cognitive composite more than one standard deviation below the mean performed low on executive function (EF), processing speed (PS), and language domains. Mean t-scores for the sample were within normal limits for total cognitive composite (μ_t-score_=55.5, p=0.08), EF (μ_t-score_=49.8, p=0.95), PS (μ_t-score_=52.5, p=0.22), language (μ_t-score_=52.8, p=0.13), and memory (μ_t-score_=57.0, p=0.01).

### Association between Lung Function and Brain Function

**Figure 2:**
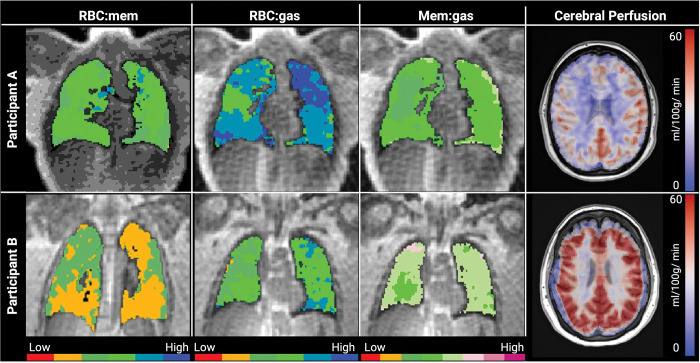
Gas exchange and ASL perfusion images Representative images of ^129^Xe gas exchange [RBC:mem, RBC:gas, mem:gas] and brain ASL perfusion overlayed onto an anatomical T1 image from a 36 year old female (Participant A) and a 58 year old female (Participant B). Functional ^129^Xe maps are colored based on standard deviation from a healthy reference distribution mean (green being normal healthy reference). Abbreviations: RBC=red blood cell; mem=membrane; ASL=arterial spin labeling

**Figure 3:**
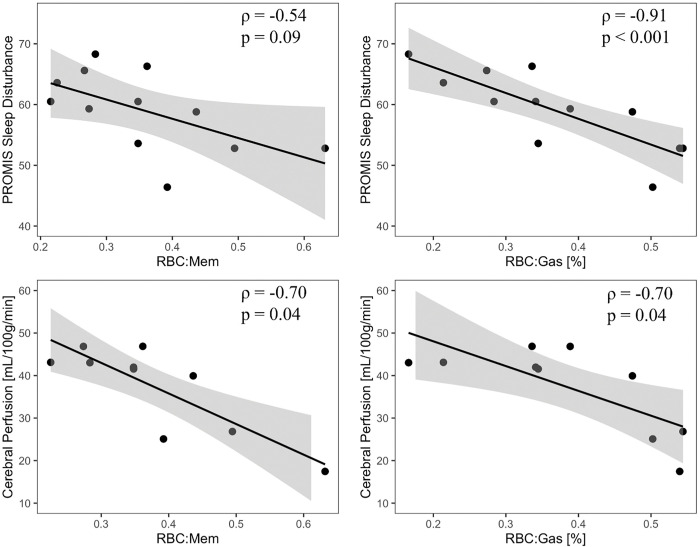
Relationships between pulmonary gas exchange, sleep disturbance, and brain perfusion *Top row:* PROMIS sleep disturbance is inversely correlated with RBC:Mem (*ρ*= −0.54, p = 0.09) and RBC:Gas (*ρ* = −0.91, p < 0.001). High PROMIS sleep disturbance scores indicate worse sleep quality. *Bottom row:* CP is inversely correlated with RBC:Mem (*ρ*=−0.699, p=0.036) and RBC:Gas (*ρ*=−0.701, p=0.035). Correlations are spearman partial correlations controlling for age. Abbreviations: RBC=red blood cell; Mem=membrane; CP=cerebral perfusion

Figure 2 shows representative lung gas exchange and ASL perfusion images from two example participants. Participant A has a higher ^129^Xe MRI measure of pulmonary gas exchange than Participant B (RBC:mem=0.49 vs 0.27), but a lower cerebral perfusion (CP; 26.8 ml/min/100g vs 46.9 ml/min/100g). Participant A also had higher objective cognitive function (t-score=65.0 vs 45.0) and lower sleep disturbances assessed via PROMIS (t-score=59.3 vs 52.8). Shown quantitatively in Figure 3, RBC:mem and RBC:gas were negatively associated with total CP measured via ASL (*ρ*=−0.70, p=0.04 for both measures). Each of which remained statistically significant when using anxiety and depression PROMIS questionnaire scores as covariates. Also in Figure 3, RBC:mem (*ρ*=−0.54, p=0.09) and RBC:gas (*ρ*=−0.91, p<0.001) were inversely correlated with severity of sleep disturbance on questionnaires. Interestingly, CP also showed a strong positive relationship with sleep disturbance (*ρ*=0.85, p=0.004).

**Figure 4:**
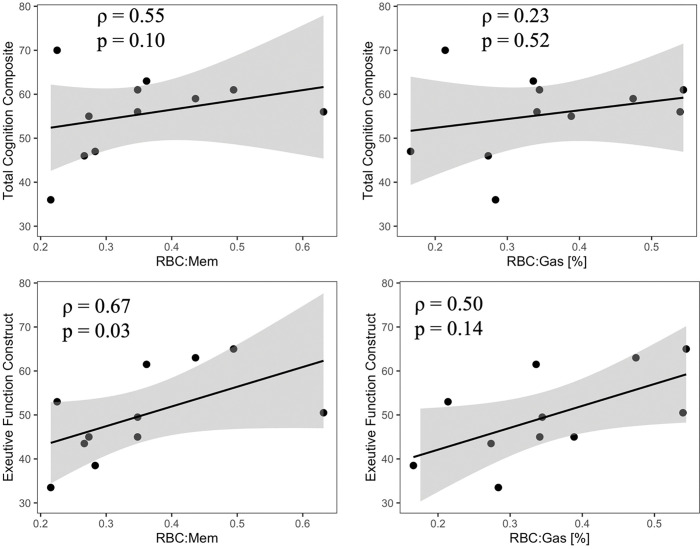
Relationships between pulmonary gas exchange and cognition *Top row*: Total cognition composite t-scores trend towards a relationship with RBC:Mem (*ρ*= 0.55, p = 0.10) but not with RBC:Gas (*ρ* = 0.23, p = 0.52) *Bottom row:* Executive function cognitive construct t-scores are correlated with RBC:Mem (*ρ* = 0.67, p = 0.03) but not with RBC:Gas (*ρ* = 0.50, p = 0.14) Correlations are spearman partial correlations controlling for age. Abbreviations: RBC=red blood cell; Mem=membrane

^129^Xe MRI measures were also associated with objective cognitive testing (Figure 4). Specifically, RBC:mem was positively correlated with EF (*ρ*=0.67, p=0.03) and showed a trend with total cognitive composite score (*ρ*=0.55, p=0.10). The associations remained statistically significant when using anxiety and depression PROMIS questionnaire scores as covariates. In contrast, ^129^Xe pulmonary MRI measures were not associated with subjective report of cognitive difficulties in everyday life (i.e., PROMIS Cognitive Function). An initial analysis of lobar brain volumes yielded significant relationships with ^129^Xe pulmonary MRI that were no longer significant after accounting for age (**Table S2**). Similarly, when evaluating WM hyperintensity volume and brain diffusion metrics (FA, MD, RD), relationships were not significant when controlling for age.

## DISCUSSION

In this investigation of 12 patients with Long COVID more than two years post-acute infection, we observed significant associations between lung function on ^129^Xe MRI, self-reported symptom surveys, quantitative cognitive performance and brain perfusion. In this study, we initially proposed three hypotheses. The most prominent findings were that reduced ^129^Xe MRI gas exchange correlated with greater sleep disturbance, increased CP via ASL, and lower performance on executive function tests, which align with the second and third hypotheses. However, contrary to the first hypothesis, these participants showed no significant differences when compared to controls. Notably, while participants reported cognitive symptoms and sleep disturbance on subjective questionnaires, their objective cognitive test scores remained within normal limits.

The discrepancy between self-reported symptoms of cognitive difficulties in everyday life and objective cognitive test performance is interesting. Some previous studies utilizing large research cohorts have documented reduced performance on objective measures of cognition including reasoning and executive function among patients with Long COVID^16–18^. But, our findings align with a body of literature reporting significant *subjective* cognitive difficulties in everyday life on questionnaire measures but average *objective* cognitive testing scores in Long COVID^19,20^. Ryan et al.^19^ suggests that cognitive effects could be subclinical and compensated for in short bursts, potentially leading to a decline in cognitive function over the course of a day that is captured by patient reported outcomes but not by brief objective tests. Whiteside et al.^20^ observed a significant relationship between cognitive complaints and psychological distress, thus concluding that mood and anxiety may be a significant contributor to perceived cognitive deficits.

Our study adds a new perspective by highlighting the potential contribution of pulmonary function and respiratory disease processes to these symptoms. Increased work of breathing may exacerbate psychological distress and the perception of poor health. Sleep disturbance is also common in people with Long COVID^19,21^ and can contribute to cognitive symptoms and mental health concerns. Poorer ^129^Xe gas exchange was correlated with elevated sleep disturbance and increased brain perfusion. Breathing difficulties can lead to poorer sleep, but we found no statistical difference between Long COVID and healthy controls with respect to PFTs or ^129^Xe MRI. Possibly pulmonary function recovery by the time of our studies well after acute infection had returned lung function back to normal, while autonomic control remained disrupted, impacting sleep or vice versa. The multifactorial nature of sleep disturbance, including stress and psychological distress, complicates efforts to establish direct causal links with cross sectional data.

Brain perfusion studies previously observed decreased cerebral perfusion in Long COVID compared to healthy controls^22,23^, similar to findings in patients with COPD where cerebral blood flow was similarly lower^24^. Our finding of a negative association between gas exchange on ^129^Xe MRI, i.e. poorer gas exchange, and brain perfusion deserves further mention and suggests the importance of brain perfusion in Long COVID. We emphasize that all brain MRI studies occurred prior to Xe MRI and so any residual subclinical anesthetic effects are not a factor. The negative association of lung function and brain perfusion could be a normal compensatory effect to offset reduced gas exchange efficiency by increasing oxygen delivery through increased blood flow to the brain. Further support of the importance of cerebral perfusion was its positive correlation with sleep disturbance. The correlation of pulmonary gas exchange and cerebral perfusion with each other and with sleep disturbance score suggest a possible common underlying mechanism such as systemic vascular injury or autonomic dysfunction contributing to both effects. A mechanism known as “fetal brain sparing”^25^ has been discovered where resting hypoxemia will lead to vasodilation in the cerebrovascular bed of a fetus and a vasoconstriction in the periphery. However, these participants did not show any signs of resting hypoxia based on finger plethysmography during Xe MRI. These complex findings underscore the need for further research in larger, well-characterized cohorts, potentially incorporating exercise or stress testing to unmask physiological deficits. Moreover, they also suggest that interventions targeting vascular regulation and sleep quality may hold promise for managing persistent Long COVID symptoms.

There are several important limitations of our study. This is a small sample, meaning it is potentially underpowered. Also, the absence of brain MRI and cognitive performance studies in our control sample for comparison with the Long COVID sample prevents evaluation of group differences. While we can show that pulmonary function is similar between the groups, it is impossible to confirm that brain perfusion differs from a healthy control sample. Therefore, we cannot determine whether these relationships are unique to the pathophysiology of Long COVID or are instead a normal compensatory mechanism of sleep disturbance or reduced pulmonary gas exchange. Moreover, it has been well established that pulmonary function and brain function change with age. Some variables in this study have reference standards (PFT, NIHTB-CB), but not the imaging metrics necessitating future comparisons to an age-matched control group. There has been recent work attempting to determine how aging and other demographic factors impact ^129^Xe gas exchange, but this work is still ongoing^26–28^. The small sample size further limits our ability to generate a multivariate model, so in this study we account for age through partial correlations.

In summary, we observed that participants with Long COVID report respiratory and cognitive symptoms, but appear normal when undergoing PFT, ^129^Xe pulmonary MRI, and objective cognitive testing greater than two years after acute infection. ^129^Xe pulmonary MRI measures of reduced gas exchange were correlated with sleep disturbance, EF, and cerebral perfusion, demonstrating a potential link between lung function and cognition that may be related to persistent symptoms from COVID-19 infection.

## METHODS

### Study Participants and Design

**Figure 5:**
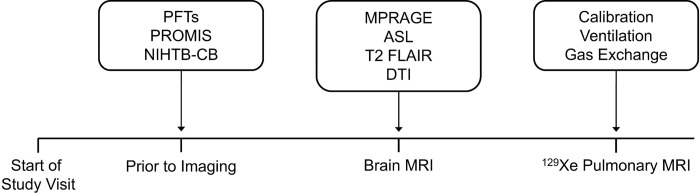
Study visit timeline Chronological order of a participant study visit. Participants began with PFT, PROMIS, and NIHTB-CB. Then brain imaging was performed, followed by ^129^Xe MRI. Abbreviations: PFT=pulmonary function testing; PROMIS=Patient-Reported Outcomes Measurement Information System; NIHTB-CB=National Institute of Health Toolbox Cognition Battery; MPRAGE=magnetization prepared rapid gradient echo; ASL=arterial spin labeling; FLAIR=fluid attenuating inversion recovery; DTI=diffusion tensor imaging; MRI=magnetic resonance imaging

Participants aged 18 years or older with persistent dyspnea and/or fatigue were recruited from a post-COVID-19 clinic at an academic medical center. Participants provided written informed consent to a protocol approved by the University of Iowa ethics board (IRB-01-202108151), which included longitudinal study visits. All experiments were performed in accordance with published guidelines, regulations and the Declaration of Helsinki. Four participants had been hospitalized during their acute infection, while the remainder were ambulatory. Inclusion criteria required a current negative nasopharyngeal swab COVID-19 PCR test and at least one of following: hospitalization during the acute infection, evidence of abnormal pulmonary function testing, or abnormalities on chest CT. Participants were excluded if there was a history of other cardiopulmonary disease or if they had any active respiratory infection. During a single study visit (Fig. 5), participants completed: symptom questionnaires, objective cognitive function testing, pulmonary function testing (PFT, including spirometry and diffusion capacity), brain MRI and ^129^Xe pulmonary MRI. Healthy controls from another study, who only underwent ^129^Xe pulmonary MRI and PFTs, were retrospectively included for comparisons. Controls underwent screening to verify no prior history of heart or lung disease and no respiratory or Long COVID symptoms. They were selected to have a similar age and sex distribution to the Long COVID sample.

### Symptoms Measures, Cognitive Function, and Pulmonary Function Testing

Participants’ medical records were reviewed to identify Long COVID symptoms from their post-COVID clinic note, as well as identify any documented comorbidities. PROMIS symptom questionnaires were administered under supervision of study personnel to assess both physical and mental health^29^. Surveys included cognitive function (v2.0, form 10a), fatigue (v1.0, form 4a), anxiety (v1.0, form 4a), depression (v1.0, form 4a), sleep disturbance (v1.0, form 4a), dyspnea severity (v1.0, form 10a), and pain interference (v1.0, form 4a). Objective cognitive functioning was assessed using the performance-based NIHTB-CB^30,31^. Both the PROMIS questionnaires and the NIHTB-CB have normative samples available to calculate a t-score relative to expectations for healthy demographically matched individuals for each questionnaire or test. The t-distribution is centered at 50 and has a standard deviation of ten, meaning that a score of 60 would indicate a performance of one standard deviation above the mean.

Age- and education-adjusted t-scores from all individual tests on NIHTB-CB were averaged to create a total cognition composite score. Domain t-score averages were also calculated including the following cognitive domains identified a priori: 1) EF (*Flanker Inhibitory Control and Attention, Dimensional Change Card Sort*), 2) PS (*Pattern Comparison Processing Speed, Oral Symbol Digit*), 3) language (*Oral Reading Recognition, Picture Vocabulary)*, and 4) memory (*Picture Sequence Memory*). Evaluation of performance validity is often conducted during cognitive assessment to identify non-credible performance due to potential insufficient effort or engagement to ensure scores are reflective of true participant ability^32^. We examined embedded validity indicators (EVI) based on NIHTB-CB scores, using a method outlined in Abeare et al.^33^. This approach was applied to all participants’ NIHTB-CB profiles using liberal cutoffs for traditional EVIs.

PFTs were performed by a certified respiratory therapist included forced expiratory volume in 1 second (FEV1), forced vital capacity (FVC), FEV1/FVC ratio, forced mid-expiratory flow (FEF25–75), and diffusion capacity of the lungs for carbon monoxide (DLCO) according to American Thoracic Society and European Respiratory Society guidelines^34,35^.

### Brain MRI Acquisition and Analysis

Brain MRI was acquired on a 3T Signa Premier (GE Healthcare, Waukesha, WI) using a 48-channel head coil with the participant supine and head-first orientation. The protocol included two structural images; a magnetization-prepared rapid gradient echo (MPRAGE) and a T2 weighted fluid-attenuated inversion recovery (FLAIR). The MPRAGE images were processed using BRAINSAutoWorkup to quantify cerebral GM and WM volumes^36^. Preprocessing was performed using Advanced Normalization Tools (ANTs) package and includes Rician denoising and an N4 bias field correction^37^. The pipeline also incorporates an iterative framework for brain parcellation adapted from Desikan-Killiany atlas^38^. FLAIR images were processed using FreeSurfer’s SAMSEG Tool to identify WM hyperintensity volume^39^.

Two additional sequences were acquired including a DTI and T1 weighted pseudo-continuous ASL scan. DTI and ASL data were processed in FMRIB Software Library^40^. For DTI, Top-up and eddy tools were used to correct for phase encoding distortion and eddy current artifacts. DTIfit was then used to reconstruct diffusion tensor models for each voxel. These tensors were used to calculate fractional anisotropy (FA), mean diffusivity (MD), and radial diffusivity (RD). The ASL acquisition included both a control and labeled image. These two images are subtracted to estimate CP. Anatomical and diffusion images were normalized to ICBM 2009b space.

### ^129^Xe MRI Acquisition and Analysis

Isotopically enriched ^129^Xe was hyperpolarized using a commercial hyperpolarizer (Model 9820, Polarean, NC). Following hyperpolarization, where the xenon was cryogenically stored, the xenon was sublimated and dispensed into a 1L Tedlar dose delivery bag. The total volume per dose was calculated using 20% of the participants predicted FVC, based on age, sex, height and ethnicity^41^. ^129^Xe MRI was acquired using the same MRI system previously mentioned for brain acquisition. ^129^Xe images were analyzed using an in-house pipeline and ANTs. Images were bias field corrected using the N4 algorithm.

The participant was positioned supine, feet-first and fitted with a commercial, single channel chest coil (Polarean Xenoview, Raleigh-Durham, NC). Three ^129^Xe scans were obtained (A calibration, ventilation, and gas exchange acquisition); each under a 10 second breath hold^42^. The calibration scan was acquired to calculate the center frequency, transmit voltage, and echo time (TE) where alveolar-capillary membrane (mem) and red blood cell (RBC) compartments are 90 ° out of phase (TE90). The ventilation acquisition is a 2D multi-slice fast gradient echo. Using an adaptive K-means clustering algorithm^43^, a ventilation defect percentage (VDP) was calculated for each participant. Both the calibration and ventilation scans used high purity nitrogen as a buffer, yielding a dose equivalent volume of 91.6 ± 9.8 mL of ^129^Xe^42^.

Gas exchange images were obtained using a 1-point Dixon technique^42,44^. These scans used an interleaved 3D radial acquisition with 1000 radial projections of each gas and dissolved phase signal were acquired with 0.5 ° and 20 ° flip angles using the previously described TE90. Gas exchange was then quantified by taking the ratio of each compartment, generating RBC:mem, RBC:gas, and Mem:gas maps and measurements. These acquisitions used unbuffered, pure xenon with a dose equivalent volume of 195.5 ± 28.6 mL of ^129^Xe. A table of all brain and pulmonary MRI metrics with their physiologic meaning is shown in Table 3.

### Statistical Analysis

R Statistical Software (V4.3.1, R Core Team, 2023) was used for statistical analysis. PFT and ^129^Xe MRI metrics were compared between Long COVID and healthy controls using Welch’s t-test. PROMIS and NIHTB-CB scores were compared to the internal reference distribution using a one sample t-test. Bivariate relationships between lung function measures and brain outcomes were assessed using Spearman partial correlations with age as a covariate. PROMIS surveys of mental health (depression and anxiety) were also included as covariates, specifically for assessment of gas exchange to executive function and gas exchange to cerebral perfusion to confirm these relationships. Specifically, correlations were performed between measures of gas exchange on ^129^Xe MRI with PROMIS symptom questionnaires (cognitive function and sleep disturbances), NIHTB-CB (total cognition composite and four cognitive domains) and brain MRI metrics (whole brain volume, WM hyperintensity volume, FA, MD, RD, and CP).

## Supplementary Material

Supplementary Files

This is a list of supplementary files associated with this preprint. Click to download.

• LongCOVIDLungBrainSupplementalInformationFinal.docx

## Figures and Tables

**Table 1.** Demographics and Pulmonary Function

Characteristic	Long COVID (n = 12)	Controls (n = 10)	P value
*Demographics*			
Age (years)	54 ± 11	41 ± 12	0.17
No. females, n (%)	10 (83)	8 (80)	0.85
BMI (kg/m^2^)	30.2 ± 7.8	273 ± 7.0	0.36
Months since infection	32 ± 5	-	-
No. hospitalized, n (%)	4 (33)	-	-
Education (years)	15 ± 2.7	-	-
*Pulmonary Function Testing*			
FEV_1_ (%_pred_)	102± 17	99 ± 12	0.62
FVC (%_pred_)	107± 15	102 ± 14	0.45
FEV_1_/FVC (%)	76 ± 1	78 ± 4	0.38
FEF_25–75%_ (%_pred_)	91 ± 28	90 ± 19	0.89
DL_CO_ (%_pred_)	96 ± 12	93 ± 6	0.57
*^129^Xe MRI*			
VDP (%)	4.0 ± 6.7	2.1 ± 2.8	0.41
RBC:Mem	0.36 ± 0.12	0.38 ± 0.08	0.57
RBC:Gas (%)	0.37 ± 0.13	0.36 ± 0.07	0.82
Mem:Gas (%)	0.98 ± 0.20	0.94 ± 0.21	0.63

Data are presented as mean ± standard deviation unless indicated otherwise. P-value from Welch’s two sample t-test. PFT only available for 8/10 healthy controls.

Abbreviations: BMI=body mass index; FEV_1_=forced expiratory volume in first second; %pred=percent of predicted value; FVC=forced vital capacity; FEF_25-75%_=forced mid-expiratory flow; DL_CO_=diffusing capacity of the lung for carbon monoxide; MRI=magnetic resonance imaging; VDP=ventilation defect percent; RBC=red blood cell; Mem=membrane

**Table 2.** Comorbidities and Long COVID Symptoms

Characteristic	Long COVID (n = 11)
*Pre-Existing Comorbidities*	
Hypertension/hypercholesterolemia	3 (27)
Asthma	3 (27)
Fibromyalgia	2 (18)
Depression	2 (18)
Neuropathy	2 (18)
Diabetes Mellitus	1 (9)
Sleep apnea	1 (9)
*Long COVID Symptoms*	
Fatigue	8 (73)
Dyspnoea	6 (55)
Headache	5 (54)
Cognitive impairment	4 (36)
Joint pain	4 (36)
Neuropathy	4 (36)
Anosmia/ageusia	3 (27)
Anxiety	3 (27)
Depression	3 (27)
Sleep impairment	3 (27)
Vision problems	3 (27)

Data are presented as n (%). Information was obtained from participants’ medical record. Pre-existing comorbidities were included if they were charted prior to COVID-19 infection. Long COVID symptoms were included from appointments relating to their Long COVID symptoms/diagnosis. Information was only available for 11/12 participants.

**Table 3.** Imaging metrics and the physiologic meaning

Metric	Physiologic Meaning
*Brain Imaging*	
MPRAGE (GM & WM Volume)	Reflects the overall brain volume. Decreased volume occurs with aging but can indicate neurodegenerative processes.
FLAIR (WM Hyperintensity Volume)	Bright spots in the WM seen in FLAIR imaging, often associated with small vessel disease and aging.
DTI (FA, MD, RD)	Measures the structure and integrity of WM tracts through the diffusion of water molecules. Can offer insight into the organization of the WM and assess myelin damage
ASL (CP)	A non-invasive imaging technique that measures brain perfusion, typically reflective of the brain's metabolic activity.
*^129^Xe Pulmonary MRI*	
VDP	The percentage of the lung that is poorly/not ventilated. High VDP can be due to obstructive or restrictive lung diseases.
RBC:mem	Measures the ratio of xenon uptake by RBCs relative to the alveolar-capillary membrane. Reflects gas transfer efficiency
RBC:gas	Measures the ratio of xenon uptake by RBCs to the xenon in the alveolar airspace.
Mem:gas	Measures the ratio of xenon uptake in the alveolar-capillary membrane relative to the xenon in the alveolar airspace. Surrogate for membrane thickness.

Abbreviations: GM=gray matter; WM=white matter; FLAIR=fluid attenuating inversion recovery; DTI=diffusion tensor imaging; FA=fractional anisotropy; MD=mean diffusivity; RD=radial diffusivity; ASL=arterial spin labeling; CP=cerebral perfusion; VDP=ventilation defect percent; RBC=red blood cell; Mem=membrane

## Data Availability

Data measures and analysis results can be made available by the corresponding author by request.
